# Chicago Pathological Society

**Published:** 1889-04

**Authors:** 


### CHICAGO PATHOLOGICAL SOCIETY.

*Stated Meeting, March 11, 1889.*

PRESIDENT I. N. DANFORTH, M.D., IN THE CHAIR.

DR. J. R. KOEHN read a paper entitled

ANTIPYRIN IN A CASE OF TYPHOID FEVER.

The patient was Mr. G., 29 years of age, parents, brothers and sisters all living and healthy. He was called to see the patient on January 6th. When 16 years of age he accidentally received a gunshot wound in the left side of the abdomen just below the spleen, exposing the gut and passing out at the back. The wound entirely healed, causing only an occasional pain. Patient had always been healthy up to the time of the accident and since, until New Year's day, when he was attacked with typhoid. Dr. Koehn ordered antipyrin to be given in ten grain doses every three hours; laudanum in ten drop doses, to be given in case of vomiting. A diet of liquid foods was prescribed, consisting of beef tea and malted milk. The night following the 12th he was called to see the patient post-haste. He found him suffering intense pain in both legs, but more in the left. Patient said he felt his feet getting cold and going to sleep, this feeling gradually extending to the knee-joint. Rubbing and turpentine fomentations soon restored the natural feeling to the right leg and foot, but the left side did not respond at all. He had a prickling sensation in the sole of the foot and sharp shooting pain in the calf of the leg. This was so intense that a hypodermic of $\frac{1}{2}$ grain morphine had not the slightest effect. The next morning temperature was a little over normal; pulse 104; heart sounds perfect; pain still intense. Inspection of the limb showed the color of the toes and sole of the foot to be of a dead white, dorsal surface of a mottled blue; no swelling. Patient said he felt as though the sole of the foot was being torn off; sensation impaired half way up the knee. Motor power almost entirely destroyed in the ankle and foot. Evening temperature on the 14th day was 100°; pulse 110. Since the morning of the 13th day the leg had been elevated on a pillow and hot fomentations applied. The foot and ankle half way up to the knee had now assumed a dull bluish red color; there was crepitation of emphysema in some parts, swelling increasing rapidly. All the signs of moist gangrene were present. The next day continuation of symptoms; temperature 103°; pulse 130, small and thready. Internal remedies had been administered from the onset of the complication. The patient was gradually growing worse. On the 19th day the temperature was 103.5°; pulse 125; patient delirious from over-stimulation. Chloral hydrate was ordered in 15-grain doses. This day the case passed out of Dr. Koehn's hands. Dr. C. Fenger, who was called in on the 22d day, found an imperfect line of demarkation, and about the condition that was present since the third day of the complication.

Auscultation gave a whizzing sound on the left side over the femoral artery, which was not heard on the right side and resembled the aneurismal thrill. Amputation was performed at the middle third of the thigh on the afternoon of the 23d day. The arteries upon being cut and tied did not exhibit the characteristic jerking with each pulsation, but were more of a pipe-stem consistency. Patient gradually grew weaker and died twenty-four hours after the operation.

Dr. Koehn in summing up, said: In searching for the cause of this complication we meet with great difficulties, as a post-mortem examination could not be obtained, and the condition of the internal organs, especially the heart, is unknown. The history of the case is free from any syphilitic taint. The remaining causes or theories that might be advanced are the following: The existence of an arterial disease described by Moxon as "inflammatory mollities," the occurrence of swelling, and softening of the arterial tunics in circumscribed spots, which become flabby and elastic. He believes that this condition depends on a peculiar state of the system and is found in young hard-working men; or, the existence of "endarteris deformans" (atheroma), which is always caused by over-exertion. The non-elastic state of the arteries in the stump shows that the large vessels above Poupart's ligament must have been diseased. Embolic plugging occurring in more peripheral vessels, as a result of detached fibrinous clots and atheromatous deposits, being washed away.

It is not impossible that one of these conditions of the circulatory system may have existed prior to the attack of the fever; the typhoid state causing impure blood, retarding the flow through the capillaries, increasing the tension of the arterial system, and thus favoring the development of the complication which caused such dire results.

Antipyrin in this case acted like magic in reducing temperature and giving the patient relief. But in causing such a profound impression on the system there is reason to believe that it also produces some important changes. Although reports in regard to serious organic changes are mostly negative, Professor Tullio reports 8 cases of articular rheumatism in which its action developed serous pericarditis, four others endo-pericarditis with subsequent mitral trouble. In another case transient albuminuria occurred, which ceased when the administration of the drug was discontinued.

Dr. C. D. Wescott read a paper entitled,

THE ACCIDENTAL COMPLICATIONS OF TYPHOID FEVER, in which he quoted considerably from Dr. Bayard Holmes' article on "Secondary Mixed Infection in Typhoid Fever". (See The Journal and Examiner, August, 1888.)

In speaking of the treatment of typhoid fever, Dr. Wescott said: The discovery of the specific microbe of typhoid fever has not, as yet, made it possible to prevent the disease, but in the light of the most recent studies, it would seem that typhoid fever, pure and simple, requires little more than careful management, and that all of the real danger to our patients is due to the secondary infections, which, possibly, we can prevent, when we fully understand them. If this can be done, may we not make typhoid fever, if not preventable, a comparatively non-fatal disease?

The treatment, *par excellence*, of the complications of typhoid is most certainly preventive, and in addition to keeping our patient as quiet and comfortable as possible, and sustaining him with proper nourishment in proper amount and at proper intervals, I think we should make him as aseptic as possible both inside and out. *Inside* by means of the intestinal antiseptics, naphthol and naphthaline, with occasional salines, unless there is exhausting diarrhœa; *externally* by means of antiseptic baths and a clean environment.

I also believe that we can do a great deal to maintain the condition of the blood and to conserve the flagging strength by the judicious administration, from the beginning, of such tonics as arseniate of strychnia, and digitaline.

In regard to antipyretics, I believe the safest we have, when their administration is understood, are *aconitine* and *veratrine;* but I do not use the chemical antipyretics myself, if I can keep my patient's temperature below 103° by resorting to tepid sponging. I certainly am not in favor of the continued and routine use of antipyrin, and believe that the chemical antipyretics, as a class, do not shorten nor materially modify the course of typhoid fever, or its intestinal lesion, and I think the largest portion of the most recent evidence fully confirms me in my opinion.

I do not think, however, that the disastrous termination of Dr. Koehn's case was due to the fact that he gave his patient antipyrin. It was probably a case of secondary infection causing thrombus, which resulted in gangrene of the leg, and the result was inevitable.

Professor A. E. Hoadly said the case reported by Dr. Koehn was one of remarkable interest; but he could not see how we could consider the condition of gangrene a complication of typhoid fever any more than any other disease. Some four weeks ago he had an elderly lady patient, who died from fatty degeneration of the heart, and the same train of symptoms were present in that case as in the one cited with moist gangrene. From the time of the onset of the pain, which marked the time of occlusion of the arteries, she lived about ten or twelve days, and he regarded it as a case of embolism. He was much pleased with Dr. Wescott's review of the complications, and the subject was one which would give the profession, when thoroughly understood, a new impetus and valuable addition to the treatment of typhoid fever. In this connection he would cite a complication of the disease which can just as well be denominated such as the case of gangrene of the leg which had been referred to.

A child, 11 years of age, was attacked with typhoid fever on the 28th of December last. About the 25th of January there was some trouble of the hip joint, which brought on neuro-muscular phenomena, fixation with flexion and adduction. The child was convalescing, and when sitting in a chair the adduction was well marked. The flexion, tenderness and pain increased, and counter-irritation was applied. Anodynes were given; the child had developed a high temperature. He was called in consultation to see what could be done for the joint disease. On examination he found the hip rounded, tense, and painful; temperature of 103.5°; pulse 130 to 135. He concluded there was suppuration of the hip joint. He gave an anæsthetic for the purpose of correcting the position of the joint. He found it was luxated. He reduced the dislocation, applied extension, and the deformity largely disappeared. There was some effusion in the joint. He waited a few days for more positive symptoms before aspirating or operating, but the temperature came down the second day to about normal. He then lightened up the weight, and adduction and dislocation again took place in spite of everything. He again administered an anæsthetic, and reduced the dislocation. He then applied a heavier weight and kept it on for a couple of weeks. The child is now sitting up, moving about, perfectly comfortable and straight. There is no swelling, no tenderness, but a moderate amount of flexion. This patient practically recovered in five weeks.

Dr. W. F. Lewis was glad some one had had a similar experience to his own, although the termination was not the same. The case he desired to report was that of a young lady who was attacked with typhoid fever, the abdomen being greatly distended, pulse of 120. He saw the patient about five weeks ago for the first time. She had previously been attended by a physician and dismissed from her first sickness about two weeks before the second illness set in. The temperature at no time arose above 103.5°. The case progressed very nicely until the 10th or 12th day, when the patient complained of pain in the ankle, calf of the leg, and in the left groin. There was very little delirium, if any, during the entire progress of the case. The first symptom complained of was pain in the left groin. Shortly afterward there was swelling, and the left limb was so flexed that it was difficult to straighten it. To alleviate the pain liniments of cotton, belladonna and camphorated soap were applied, after which it gradually abated. When the swelling had become quite tense, he applied the faradic current, and after the first application there was a modification of the pain. Two more electrical applications were made, when there was scarcely any pain experienced. On the fourth day from the time of the commencement of the faradic current, the swelling had entirely disappeared, and the limb was then easily straightened. He would like to inquire whether any of the members had used the continuous or interrupted current in such cases.

Dr. Koehn said the faradic current had been used (but not persistently) in his case for a few days without any beneficial result.

Professor H. M. Lyman called attention to the extensive field covered by typhoid lesions. It is a rare thing to see very many of the complications appear in the same individual, but if we take a large number of cases of the disease, hardly any part of the body could be found that had not been, or would not be, involved as a complication. He would call attention more particularly to the manner in which the nervous system is sometimes complicated as a consequence of the disease. And there were two ways in which the nervous system might become influenced by it. In the first place, we may have the direct poisonous effect of the typhoid bacillus and its excreta upon the brain, the spinal cord, and the nervous apparatuses of the body. This usually manifests itself in the ordinary course of the disease. Then later, after the typhoid fever proper has run its course, we may have complications involving the same organization, which, however, are of a somewhat different character. For example, we may have, as a complication, the brain affected in the early stage of the fever and during its course, which resembles more the effects of a subacute meningitis than what would be observed later. Post-mortem examination of patients dying in this condition shows an exudation along the walls of the vessels. There is a somewhat increased quantity of cerebro-spinal fluid, and a slight turbidity of the membranes. But when the complication occurs late (after the typhoid poison itself has run its course, and perhaps has been eliminated from the body) we may have disorder of the brain consequent upon an impoverished condition of the blood or upon an exhausted state of the brain and nervous system. In the late stage we may have a form of meningitis which involves the membranes of the brain and spinal cord in such a way as to considerably resemble the phenomena of cerebro-spinal meningitis. These phenomena (which present themselves as complications in typhoid fever) do not exhibit themselves in the early stages of the disease. They vary according to the period of their manifestation.

In the spinal cord we may have complications, and these may take the character of a somewhat acute form of meningitis, or of chronic degenerations of the spinal cord itself, producing the various phenomena of muscular paralysis and atrophy consequent upon disease of the trophic centers in the grey matter of the spinal cord.

Then, lastly, we may have peripheral inflammations involving the nerves themselves, so that the patient will manifest loss of sensibility and power of motion in certain parts of the body, according to the diffusion of the inflammatory condition involving the nerves. These cases, of course, are rare, but they do occur after typhoid fever just as they occur after any fever that is produced by toxæmia. They may be observed after scarlet fever, measles, diphtheria not unfrequently and after small-pox. These are the principal complications that involve the nervous system. They are acute and resemble the more common forms of inflammation during the time of operation of the typhoid poison, but resemble more the forms of chronic degeneration of the brain, spinal cord and nerves, trunks and branches when they occur late, after the typhoid fever has expended its force.

Dr. J. E. Colburn said that some years ago when he was in general practice in northern New York quinine was used in large doses, and as a result ear complications developed; in some cases there was more or less complete deafness, and this was attributed very largely to the excessive use of the drug. He understands it is not used so extensively now, and that other remedies are used in its place. He would like to ask if the same frequency of deafness occurs through the use of other remedies, where there is no marked brain lesion or nervous organic lesion following. The quinine undoubtedly has something to do with the production of the deafness where it is used extensively, and he would like to know whether the same results followed its use in typhoid fever.

Recently, he had observed a case in which neuro-retinitis followed upon a typhoid condition within three weeks. He saw the patient at her home. In this case, during and immediately subsequent to the fever, there were no symptoms of marked brain lesion, delirium, or disturbance, except the retinitis, which was very marked, and ran a rather acute course, terminating apparently without very much atrophy.

Dr. A. Hinde had seen neuro-retinitis follow two or three cases of typhoid fever. The cases were observed in the convalescing period. In reply to Dr. Colburn, who asked whether there were any nervous symptoms observed during the fever, Dr. Hinde said that he did not see the cases during the fever.

In reply to Dr. John D. Skeer, who asked how much quinine was given, Dr. Colburn said he had not given any. He had only treated the neuro-retinitis present.

Professor Hoadley had a case to report in this connection. The patient, 13 years of age, in the second week of typhoid fever, became absolutely deaf, this condition continuing some three weeks after convalescence had been established. There was no quinine given. The deafness came on gradually. There was no nervous disturbance or brain lesion. It was a very mild case of typhoid and ran a pleasant course. The patient fully recovered from the deafness in about six weeks from the time it commenced.

Dr. Blinn reported the case of a boy, 3½ years old, who came under her observation two years ago. A sister had died of typhoid fever, and another brother had been ill with the disease, but recovered. The patient was sick about five weeks. After the second week he became blind, deaf, dumb, and was greatly emaciated. There was very little hope of recovery, but in the course of three weeks from the time he began to lose his senses, he slowly and completely recovered, so that now he is bright and healthy.

The President said he was called to see the case reported by Dr. Koehn, and made the suggestion that possibly the gangrene of the leg may have been due to antipyrin. He ventured this suggestion because his experience with the drug in St. Luke's Hospital had been very unfavorable. The internes of the institution had given it in from 15 to 20 grains. When the temperature arose above a certain point—perhaps 102 or thereabouts—antipyrin was administered to force it down, and in two or three cases alarming depression followed its use. He had been cautious in its use since that time for two reasons: (1) Because of the consequent cardiac failure, and (2) because he was convinced that, so far as the typhoid fever went, it did not do any good in either shortening the disease, or modifying the lesions consequent upon it. He thought it was not improbable that there had been a venous thrombus due to cardiac failure.

A few days after he was called to see Dr. Koehn's case he went East, and while in a doctor's office in Boston he picked up a copy of the *Boston Medical and Surgical Journal*, and saw an account of a similar case in which gangrene of the left leg had followed typhoid fever. Amputation was performed, and the patient recovered. There was no account given in the journal of an examination of the vessels of the leg, nor was there any theory advanced as to the cause of it. The writer, however, called attention to similar cases cited in Pepper's System of Medicine, in the article by Hutchinson, of Philadelphia. If the patient's leg had been preserved and examined it would either substantiate or overthrow the bacillus theory. He was not, as yet, an absolute convert to the so-called microbic theory of disease.

Dr. J. M. Patton wished to speak of antipyrin as possibly causing the condition found in the case reported by Dr. Koehn. It recalled a case of typhoid fever that he had treated two or three years ago when antipyrin was first introduced as a remedy in the treatment of the disease. The patient, a married woman, 22 years of age, was in good health up to the time of the attack. He had used all the antipyretics he could think of without bringing the temperature below 102°. Quinine the patient could not tolerate, either the sulphate or bromide, so he used antipyrin. The foreign journals were then recommending it in gramme doses, to be administered in quick succession. He gave the patient three gramme doses the next day, and about four o'clock in the afternoon the husband came for him very much frightened, and said his wife was going to die. Dr. Patton found the patient in a rather peculiar condition, the whole surface of the body being of a red hue, which was produced by dilatation of the capillary vessels. The patient was in a nervous condition. He assured the relatives and friends she would be all right. He remained to await developments. The temperature when the antipyrin was given was 103°; at the time he saw her it was about 99½°. He remained with the patient about two hours, when the natural color returned, and the temperature arose to 104° before he left the house. The next day the patient was very much depressed, and developed a nervous twitching about the muscles of the mouth, which she was unable to control. The thought at the time occurred to him that antipyrin administered to persons of her constitution in typhoid fever was a dangerous remedy. If the drug has such an effect on the vessels, he could not see why it might not produce such a condition. Whether a thrombus is indirectly due to microbes or ptomaines, we must necessarily have some local condition, otherwise we should have thrombosis in all cases of severe poisoning by microbes, and it seemed to him that antipyrin may (by dilating the vessels and changing the blood pressure as it did in this instance) furnish the conditions necessary for a thrombus to develop.

Dr. Wescott, in closing the discussion, said that Bristow and other authorities mention thrombosis causing gangrene of the leg, following or complicating typhoid fever even before antipyrin was given. He thought the theory was established, as well as anything could be established in bacteriology, that there is a specific bacillus which with its ptomaine so influences the human body as to give us the collection of symptoms that we call typhoid fever.

Dr. L. B. Hayman read a paper on the

HYPODERMIC USE OF PILOCARPINE IN SEROUS EFFUSIONS, in which he said he had used pilocarpine in a number of instances, both as a galactagogue and as a diaphoretic, and in one case the use of the drug was continued for several months with most gratifying results.

*Case.*—The patient was a young lady of 17 who came to the city from Earlville, Illinois, suffering from chronic Bright's disease. She was under the care of Professor I. N. Danforth, who, upon leaving the city for a time, placed her in my charge, and I saw her for the first time on the 5th of September, 1887. She had been sick for about eight months, and was in the later stages of chronic nephritis. There was extreme œdema of all the cellular tissues of the body, so that the patient could not move from her chair. Both pleural sacs contained fluid, the respiration was difficult and there was a harsh dry cough; the heart was labored in its action. For days the patient had been unable to lie down and spent the nights in her chair, catching an occasional short nap with her head resting upon a pillow on her lap. She was restless and nervous, although very patient. But little nourishment could be retained, and the end was apparently so near that relatives had been summoned.

Diaphoresis had been invited by hot air baths, and diuretics and hydragogue cathartics had been freely used; but still the dropsy increased. The swollen feet had been tapped twice, allowing of considerable escape of serum, with but temporary benefit.

An examination of the scanty urine showed an immense deposit of albumen, about three-fourths of the contents of the test tube solidifying with heat. The urine was of high specific gravity, and showed under the microscope a large number of blood corpuscles, clouds of granular and epithelial casts, with renal epithelium.

The weak, rapid heart, and the labored respiration both contra-indicated the use of pilocarpine, but other remedies had failed and it offered the only chance of benefit, so I decided to use it. The patient was placed in bed—seated, for she could not lie—and a hot air apparatus was placed under the blankets. She was given half an ounce of brandy, and then a hypodermic injection of $\frac{1}{3}$ of a grain of the muriate of pilocarpine. In about a minute the constitutional effects of the drug appeared, and soon there was an abundant secretion of saliva and the attendants were busy wiping the streams of perspiration from her face and neck. There was coughing with free expectoration of mucus; the respirations became easier, and the pulse, which had been about 120 to the minute, fell to 100.

The patient expressed herself as wonderfully relieved. The perspiration lasted about two hours and was followed by a quiet night. The injection was repeated on the following day with similar results. In each case the sweating was profuse and was followed by a marked diminution in the nervous symptoms. The injections were given daily for several weeks, accompanied by iron, digitalis and hydragogue cathartics. Under this treatment the dropsy steadily diminished, so that soon the patient could lie down; the nervousness was lessened, and there was a gain in appetite and strength. The urine was also improved.

The amount of albumen was much diminished and the casts, at first so numerous, were found with difficulty. There seemed to be real improvement, not only in the general symptoms, but in the condition of the kidney as well,

The gain was so gratifying that, after about four weeks, the patient, feeling sure of ultimate recovery, began to speak of returning home. She thought the treatment might still be carried on at her home in Earlville, and on the 5th of October, contrary to advice, she took the train for home.

Because of the condition of the heart, her home physician, Dr. E. T. Goble, of Earlville, Illinois, would not use the pilocarpine until after he had communicated with me, and in the two or more weeks which intervened the dropsy rapidly returned. The hypodermics were then recommenced, and persisted in almost daily until her death, which occurred on the 26th day of May following, about eight months later.

The pilocarpine seemed always to benefit, and the patient would plead for the hypodermics. They always relieved the nervousness,—probably by the elimnation of urea, quieted and slowed the heart and gave ease and comfort. It is quite certain in this case, that while it did not avert the fatal issue, the use of the drug gave comfort, and prolonged life for many months.

